# Reduction responsive and surface charge switchable polyurethane micelles with acid cleavable crosslinks for intracellular drug delivery†

**DOI:** 10.1039/c8ra01581c

**Published:** 2018-05-16

**Authors:** Lili Zhao, Chang Liu, Zhuangzhuang Qiao, Yan Yao, Jianbin Luo

**Affiliations:** College of Chemistry and Environmental Protection Engineering, Southwest Minzu University 610041 Chengdu China luojb1971@163.com +86 28 85522269

## Abstract

Previously we synthesized redox sensitive polyurethane micelles, core crosslinked by diisocyanates (PU-CCL). To improve the intracellular drug release and tumor cellular toxicity of anticancer drugs loaded into polyurethane micelles, we now describe redox sensitive polyurethane micelles with tunable surface charge switchabilities, crosslinked with pH cleavable Schiff bonds, as anticancer drug carriers. Different amounts of 1,6-diaminohexane were connected onto the pendant carboxyl groups of amphiphilic multi-blocked polyurethane (PU-SS-COOH), resulting in polyurethanes with various ratios of pendant carboxyl and amine groups (denoted as PU-SS-COOH-NH_2_-1, PU-SS-COOH-NH_2_-2 and PU-SS-COOH-NH_2_-3). The surface charge switched as the pH was increased for PU-SS-COOH-NH_2_-1, PU-SS-COOH-NH_2_-2 and PU-SS-COOH-NH_2_-3. Then the PU-SS-COOH-NH_2_-3 micelles, dissolved in water, were crosslinked by glutaraldehyde resulting in surface charge switchable and reduction responsive polyurethane micelles with acid cleavable crosslinks (PU-ACCL). The crosslinked polyurethane micelles (PU-ACCL) demonstrated superior particle stability in phosphate buffered saline (PBS, pH = 7.4) solution without reducing agents, whereas the drug release rate was markedly accelerated by the addition of glutathione (GSH). Notably, the drug release from PU-ACCL was further accelerated in acidic fluid as the result of acid induced cleavage of the crosslinks. *In vitro* cytotoxicity studies demonstrated that doxorubicin (DOX)-loaded PU-ACCL micelles displayed increased cytotoxicity against tumor cells which was comparable to that obtained for DOX loaded into uncrosslinked polyurethane micelles. The reduction responsive and surface charge switchable polyurethane micelles with acid cleavable crosslinks, which have superior extracellular stability and provide rapid intracellular drug release, may hold great potential as a bio-triggered drug delivery system for cancer therapy.

## Abstract 2

Previously we synthesized redox sensitive polyurethane micelles, core crosslinked by diisocyanates (PU-CCL).

## Graphical abstract

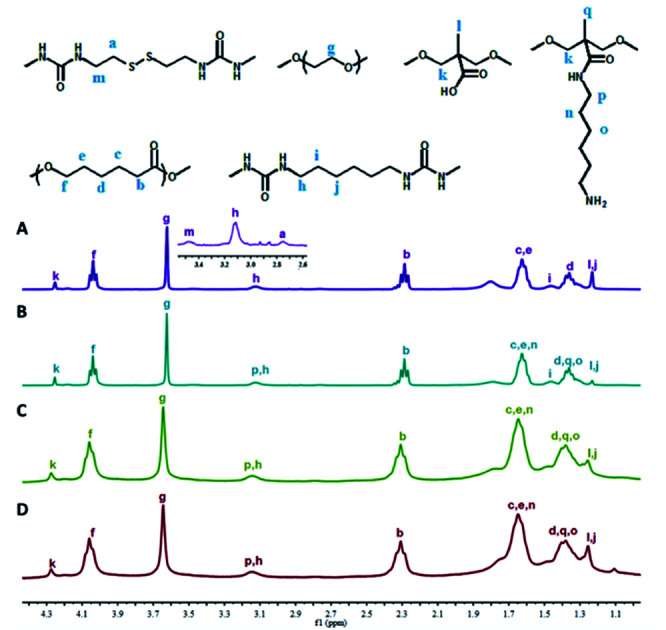

## 1. Introduction

**Scheme 1:**
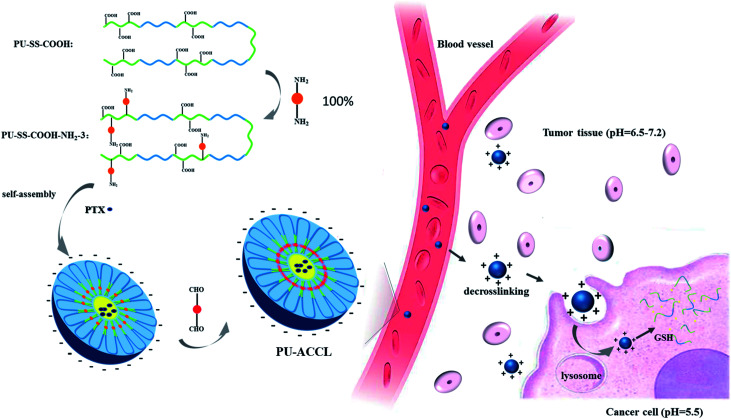
Schematic illustration of the reduction sensitive polyurethane with carboxyl groups (PU-SS-COOH), and amine groups (PU-SS-COOH-NH_2_); the preparation of crosslinked PU-ACCL; and the self-assembled PTX-loaded micelles as effective intracellular drug delivery nanocarriers.

Stimuli-responsive self-assembled micelles based on amphiphilic blocked copolymers or surfactants have been developed as drug carriers in recent years, to encapsulate and protect hydrophobic drugs from premature release and immune clearance.^1–6^ The release of the payloads from these responsive micelles happens in a controlled manner on arrival at the target site, only when triggered by external stimuli, such as light, temperature, pH, redox potential, enzymes and ionic strength.^7–12^ Recently, multi-responsive assemblies have been developed to optimize the release of functional species in different environments. Biodegradable multi-segmented polyurethanes (PUs) with stimuli responsive properties have been widely studied as drug carriers due to their good biocompatibilities and tailorable molecular structures.^13–18^ To prevent the premature release caused by rapid dilution after intravenous administration, and to resolve the surface charge dilemmas of long circulation *versus* cellular uptake, we previously prepared surface charge switchable PU micelles with reduction responsive properties which were core crosslinked by diisocyanates (PU-CCL).^19^ However, two main problems still existed in our previous design. Firstly, the pH value for the surface charge transformation was at ∼4.70 which is not appropriate for anticancer drug delivery. Therefore, the first objective of this study was to prepare PU micelles with a tunable surface charge switching point by introducing various amounts of amine groups. Secondly, the uncleavable urea bonds formed by the reaction of diisocyanates with pendant amine groups resulted in slow and insufficient drug release which led to the lower tumor cellular toxicity of drugs encapsulated in PU-CCL micelles when compared to drugs encapsulated in their uncrosslinked counterparts. Therefore, the second aim of this study was to use glutaraldehyde as a crosslinker by reacting it with primary amine groups, to form pH sensitive Schiff base bonds, and so to synthesize core crosslinked PU micelles with redox and pH responsive properties and enhanced drug release efficiency (PU-ACCL). The Schiff's base is relatively stable in physiological conditions (neutral), while it can be considered to be completely decomposed at a pH value below 6.5.^20–23^ Moreover, the primary amine groups are formed again in an acidic microenvironment after the cleavage of the Schiff base bonds which may facilitate the cellular uptake of PU micelles and the endosome escape process. Therefore, it is reasonable to expect an enhanced anticancer activity of DOX when loaded into glutaraldehyde crosslinked PU micelles (PU-ACCL). To the best of our knowledge, redox sensitive PU micelles crosslinked by pH labile Schiff base bonds have not been reported previously (Scheme 1).

## 2. Experimental

### 2.1 Materials

Poly(ε-caprolactone)diol (PCL *M*_n_ = 2000, Dow Chemical, USA) and polyethylene glycol (PEG) (*M*_n_ = 1000, Sunshine Biotechnology (Nanjing) Co., Ltd., China) were dehydrated under reduced pressure at 100 °C for 2–3 h before use. *N*,*N*-Dimethylacetamide (DMAc) was dried over CaH_2_ and vacuum distilled before use. Dibutyltin dilaurate and hexamethylene-1,6-diisocyanate (HDI) were obtained from Aladdin Industrial Corporation, China, and used without further purification. Triethylamine (TEA, Aladdin Industrial Corporation, China) was distilled under vacuum. Cystamine dihydrochloride (Cys, Aladdin Industrial Corporation, China), 2,2-bis(hydroxymethyl)propionic acid (DMPA) and dicyclohexylcarbodiimide (DCC) were used as received. Human Umbilical Vein Endothelial Cells (HUVECs) and HepG2 cells were purchased from Procell (Wuhan). Doxorubicin hydrochloride (DOX, –NH_3_^+^Cl^−^ salt form, >98%) and Paclitaxel (PTX, 99.5%) were purchased from AstaTech (Chengdu) Pharmaceutical Co. Ltd., China. Sodium dodecyl sulphate (SDS) and glutathione, glutaraldehyde (25%) were obtained from Kelong Chemical Reagent Factory, Chengdu, China.

### 2.2 Synthesis of polyurethanes

Using the traditional two step solution polymerization process that we reported previously, PU with pendant carboxyl groups and disulfide linkages on the main chain (PU-SS-COOH) was synthesized from PEG, PCL, HDI, Cys and DMPA.^19^ PUs with various numbers of pendant amine groups (PU-SS-COOH-NH_2_) were synthesized by a DCC condensation reaction between the pendant carboxyl groups and 1,6-diaminohexane with different –COOH/NH_2_ feed ratios, as reported previously. The feed ratios are listed in Table 1.

**Table 1:** Feed ratios and molecular weights of PU-SS-COOH, PU-SS-NH_2_-1, PU-SS-NH_2_-2 and PU-SS-NH_2_-3

Samples	Feed ratio (mol)					Feed molar ratio of (COOH/NH_2_)	Resultant molar ratio of (COOH/NH_2_)	Molecular weights (g mol)		
	PCL	PEG	HDI	DMPA	Cys			*M*_w_	*M*_n_	*M*_w_/*M*_n_
PU-SS-COOH	3	1	8.1	3	1			23 200	13 200	1.758
PU-SS-COOH-NH_2_-1	3	1	8.1	3	1	1 : 0.8	1 : 0.7	24 800	13 200	1.879
PU-SS-COOH-NH_2_-2	3	1	8.1	3	1	1 : 1	1 : 0.9	25 800	18 100	1.425
PU-SS-COOH-NH_2_-3	3	1	8.1	3	1	1 : 2	1 : 1	28 000	18 900	1.481

### 2.3 Characterization of polyurethanes

^1^H NMR spectra were recorded on an Agilent NMR VNMRS 400 (400 MHz) spectrometer using deuterated chloroform (CDCl_3_) as the solvent. The molecular weights and molecular weight distributions of PU-SS-COOH, PU-SS-COOH-NH_2_-1, PU-SS-COOH-NH_2_-2 and PU-SS-COOH-NH_2_-3 were determined and recorded using Gel Permeation Chromatography (GPC, Waters-1515). *N*,*N*-Dimethylformamide (DMF)/LiBr was used as the mobile phase with a flow rate of 1 mL min^−1^ at 40 °C; the molecular weights are reported relative to polystyrene (PS) standards.

### 2.4 Preparation of polyurethane micelles

A dialysis method was used to prepare the PU micelles. PU samples (PU-SS-COOH, PU-SS-COOH-NH_2_-1, PU-SS-COOH-NH_2_-2 and PU-SS-COOH-NH_2_-3, 20 mg) were completely dissolved in 2 mL DMAc. Then, each solution was added dropwise into 10 mL distilled water with Na_2_HPO_4_·12H_2_O (0.2 mol L^−1^) under vigorous stirring. To prepare the PU-ACCL micelles, PU-SS-COOH-NH_2_-3 (20 mg) was completely dissolved in 2 mL DMAc. Then, the solution was added dropwise into 10 mL distilled water with Na_2_HPO_4_·12H_2_O (0.2 mol L^−1^) and glutaraldehyde (25%, 10 μL) under vigorous stirring. The reaction mixture was reacted at room temperature for 24 h with stirring. Subsequently, each micelle solution was moved to a dialysis bag (MWCO: 3.5 kDa) and dialysed against distilled water (pH = 8) for 72 h to eliminate the organic solvent at room temperature. The micelle solutions were filtered by passing through a 0.45 μm pore-sized syringe filter (Millipore, Carrigtwohill, Co. Cork, Ireland), and stored at 4 °C.

### 2.5 Characterization of polyurethane micelles

The critical micelle concentration (CMC) of the micelles was determined using pyrene as a fluorescent probe. The concentration of PU was varied from 1.0 × 10^−5^ to 0.2 mg mL^−1^ and the final pyrene concentration was fixed to 5.2 × 10^−6^ M. The combined solution of pyrene and micelles was sonicated for 4 h in the dark before the fluorescence measurement. The fluorescence spectra were determined using fluorometry (Varian Cary Eclipse Fluorescence Spectrophotometer, Agilent, USA) over a wavelength range from 285 to 355 nm, with the emission wavelength at 372 nm and with 5 nm slits for both excitation and emission. The CMC was estimated as the cross-point when extrapolating the intensity ratio *I*_337_/*I*_333.5_ at low and high concentration regions.

The size and zeta potentials of the nanoparticles in aqueous solution were measured by a Zetasizer analyzer (Malvern Zetasizer Nano, Zen 3690+MPT2, Malvern, UK). The kinetic stabilities of the PU-SS-COOH, PU-SS-COOH-NH_2_-3 and PU-ACCL micelles in deionized water were studied in the presence of SDS as a destabilizing agent. The micelle solutions (1 mg mL^−1^) were mixed with SDS aqueous solution (20 mg mL^−1^) in a 2 : 1 v/v ratio and the scattered light intensity was monitored by dynamic light scattering (DLS) over a period of 48 h.^24^ The morphologies of PU-SS-COOH, PU-SS-COOH-NH_2_-3 and PU-ACCL micelles were observed by transmission electron microscopy (TEM, Tecnai G2F20 transmission electron microscope, Royal Dutch Philips Electronics Ltd., Holland) at an accelerating voltage of 200 kV with phosphotungstic acid 2% negative staining.

### 2.6 Preparation of PTX-loaded micelles

To prepare PTX-loaded PU-SS-COOH and PU-SS-COOH-NH_2_-3 micelles, a clear solution consisting of the purified, dried PU (10 mg) and PTX (5 mg) in DMAc (1 mL) was added dropwise to deionized water (10 mL). To prepare the PTX-loaded PU-ACCL micelles, PU-SS-COOH-NH_2_-3 (10 mg) and PTX (5 mg) were dissolved in 2 mL DMAc. Afterwards, the solution was added dropwise into 10 mL distilled water with Na_2_HPO_4_·12H_2_O (0.03 mol L^−1^) and 10 μL glutaraldehyde (25%) under vigorous stirring. The reaction mixture was reacted at room temperature for 24 h with stirring. Subsequently, the micelle solution was moved to a dialysis bag (MWCO: 3.5 kDa) and dialysed against distilled water (pH = 8) for 72 h at room temperature to eliminate the organic solvent. The micelle solution was passed through a 0.45 μm pore-sized syringe filter (Millipore, Carrigtwohill, Co. Cork, Ireland), and stored at 4 °C. To evaluate the concentration of the drugs in loaded micelles, a high-performance liquid chromatography (HPLC) system (Waters Isocratic HPLC Pump, US) equipped with a reverse-phase C18 column (4.6 × 250 mm, 5 μm) was used. The mobile phase was acetonitrile-water (60/40 v/v) and the flow rate was 1.0 mL min^−1^. The UV absorption of PTX in the flow was recorded by a Waters 2489 UV/visible detector at a wavelength of 227 nm. The encapsulation efficiency (EE%) and loading content (LC%) were calculated based on the equations below:LC (%) = *W*_e_/*W*_m_ × 100%where *W*_e_ is the mass of the drugs in micelles; and *W*_m_ is the total mass of the loaded micelles.EE (%) = *W*_e_/*W*_f_ × 100%where *W*_e_ is the mass of the drugs in micelles; and *W*_f_ is the initial amount of drugs in the feed.

### 2.7 Preparation of DOX-loaded micelles using the dialysis method

Water (5 mL) was added dropwise into a clear solution consisting of the purified, dried PU (10 mg), DOX (2 mg), and Et_3_N (3 molar equivalents to DOX) in DMF (2 mL). The resulting dispersion was dialyzed against water (500 mL) for 2 days, yielding DOX-loaded micelles of PU at 1.0 mg mL^−1^. The excess DOX was removed by passing the sample through a 0.45 μm filter. The concentration of the drugs in the loaded micelles was evaluated by using UV/vis spectrophotometry (UnicamUA500, Thermo Electron Corporation). The UV absorption of DOX was determined at a wavelength of 482 nm. The LC (%) and EE (%) were calculated based on the equations shown in Section 2.6.

### 2.8 *In vitro* release of PTX

*In vitro*, drug release from the drug-loaded micelles was performed by using the dialysis method. In brief, 2.5 mL drug-loaded micelle solution (PU-SS-COOH, PU-SS-COOH-NH_2_, PU-ACCL) was added to a dialysis bag (MWCO: 8000) which was then immersed in 25 mL of PBS (0.01 M, pH 7.4, pH 6.8 or pH 5.5) containing 0.1 M sodium salicylate, with or without 10 mM GSH. The dialysis system was kept at 37 °C in a thermostatic incubator with a shaking speed of 110 rpm. Samples were taken out and replaced with the same volume of fresh medium at desired time intervals. HPLC was used to analysis the concentration of PTX released from the drug-loaded micelles. The cumulative percent drug release (*Q*) was calculated using the equation below:*Q* (%) = *M*_x_/*M*_y_ × 100%where *M*_x_ is the cumulative weight of drug released; and *M*_y_ is the mass of the drug in the micelles.

### 2.9 Cell culture

Human liver cell line HepG2 was cultured in RPMI 1640 medium supplemented with 2 mM l-glutamine, 100 U mL^−1^ penicillin and 10% fetal bovine serum (FBS) (HyClone, Logan, UT) at 37 °C in a humidified atmosphere containing 5% CO_2_ (Sanyo Incubator, MCO-18AIC, Japan).

HUVECs were maintained in Dulbecco's modified Eagle's medium (DMEM, Gibco Life, Grand Island, NY, USA) supplemented with 10% (v/v) FBS (HyClone, Logan, UT), 2 mM l-glutamine and 1% (v/v) antibiotics mixture (10 000 U of penicillin and 10 mg of streptomycin) (Gibco). The cells were incubated in a humidified atmosphere of 5% CO_2_ at 37 °C (Sanyo Incubator, MCO-18AIC, Japan).

### 2.10 Cellular uptake and intracellular release of payloads

The intracellular trafficking of DOX was observed in HepG2 cells by using confocal laser scanning microscopy (CLSM). The DOX-loaded micelles were incubated with HepG2 cells for 1 h at 37 °C. After removal of the medium, the cells were washed with cold PBS, fixed with 1 mL of 4% paraformaldehyde for 30 min at 4 °C, and stained with 2-(4-amidinophenyl)-6-indolecarbamidine dihydrochloride (DAPI, Roche) for 10 min. The washing procedure was repeated three times. Finally, the slides were mounted with 10% glycerol solution then observed using a LeicaTCS SP8 microscope (Leica Microscopy Systems Ltd., Germany).

### 2.11 Cell viability assay

HepG2 cells and HUVECs were seeded in 96-well plates at 4 × 10^3^ cells per well and incubated for 24 h. In order to evaluate the antitumor activity of the PTX-loaded PU micelles and cytocompatibility of the drug-free micelles,^25^ the culture medium was removed and replaced with 100 mL medium containing various concentrations of micelle solutions for another 24 h of incubation. Next, 10 μm of Cell Counting Kit-8 (CCK-8) solution (100 T mL^−1^, Shanghai Qcbio Science & Technologies Co., Ltd.) was added to each well. After incubating the cells for 4 h, the absorbance was measured at a wavelength of 450 nm. The cell viability was normalized to that of cells cultured in the full culture medium. Dose-effect curves were plotted and the median inhibitory concentration (IC_50_) was determined using the software IBM SPSS Statistics (SPPS, Inc., USA).

## 3. Results and discussion

### 3.1 Synthesis and characterization of polyurethanes

The unimodal GPC curves of purified PU-SS-COOH, PU-SS-COOH-NH_2_-1, PU-SS-COOH-NH_2_-2 and PU-SS-COOH-NH_2_-3 (Fig. S1†) confirmed that the polymerization to make the PUs was successful. Notably, the retention times of PU-SS-COOH-NH_2_-1, PU-SS-COOH-NH_2_-2 and PU-SS-COOH-NH_2_-3 (Fig. S1†) were a little shorter than that of PU-SS-COOH, suggesting the successful grafting of 1,6-diaminohexane. The average molecular weights and polydispersities of PU-SS-COOH and PU-SS-COOH-NH_2_ are listed in Table 1.

**Fig. 1:**
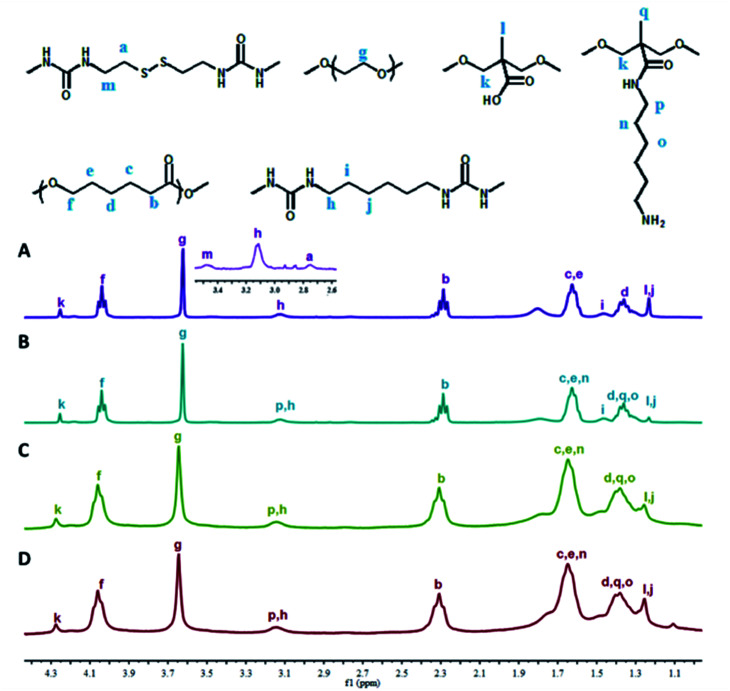
^1^H NMR spectra of PU-SS-COOH (A), PU-SS-COOH-NH_2_-1 (B), PU-SS-COOH-NH_2_-2 (C) and PU-SS-COOH-NH_2_-3 (D) in CDCl_3_.

Fig. 1 shows the representative ^1^H NMR spectra of PU-SS-COOH, PU-SS-COOH-NH_2_-1, PU-SS-COOH-NH_2_-2 and PU-SS-COOH-NH_2_-3, and the assignment of the peaks. Except for the presence of the resonance peak corresponding to diaminohexane in the ^1^H NMR spectra of the PU-SS-COOH-NH_2_ samples, the ^1^H NMR spectra of PU-SS-COOH-NH_2_-1, PU-SS-COOH-NH_2_-2 and PU-SS-COOH-NH_2_-3 are nearly identical, arising from their similar compositions. The resonance peaks centred at 4.04 ppm (peak f), 2.29 ppm (peak b), 1.62 ppm (peaks c and e) and 1.36 ppm (peak d) are attributed to the protons of the PCL units, while the peak at 3.62 ppm (peak g) is assigned to the protons on the PEG units. The resonances corresponding to the methylene protons of the HDI units in the products are located at 3.12 ppm (peak h), 1.46 ppm (peak i) and 1.22 ppm (peak j). The presence of the methylene protons of the Cys units leads to peaks at 3.46 ppm (peak m) and 2.75 ppm (peak a) suggesting the successful incorporation of Cys in the resulting PUs. The presence of DMPA units are proven by the presence of the resonances at 4.25 ppm (peak k) and 1.22 ppm (peak l) corresponding to the methylene and methyl protons of the DMPA, and indicating the presence of the carboxyl groups in the PU-SS-COOH. The presence of resonances at 3.12 ppm (peak p), 1.62 ppm (peak n) and 1.36 ppm (peak o), corresponding to the methyl protons of the diaminohexane units in PU-SS-COOH-NH_2_-1, PU-SS-COOH-NH_2_-2 and PU-SS-COOH-NH_2_-3, indicate the successful attachment of diaminohexane onto the carboxyl groups of PU-SS-COOH, and the presence of primary amine groups in the PU-SS-COOH-NH_2_-1, PU-SS-COOH-NH_2_-2 and PU-SS-COOH-NH_2_-3. The amount of diaminohexane grafted onto the pendant carboxyl groups (molar%) was calculated based on the peak area ratio of peak k to peak p (the methyl protons of DMPA and diaminohexane, respectively). The grafting ratio (%) = 2(peak area of peak p)/(peak area of peak k) × 100%. About 71, 90 and 100 molar% carboxyl groups were grafted by diaminohexane for PU-SS-COOH-NH_2_-1, PU-SS-COOH-NH_2_-2 and PU-SS-COOH-NH_2_-3, respectively. Therefore, the molar ratio of primary amine to carboxyl groups is 9/1 in PU-SS-COOH-NH_2_-2. The presence of primary amine groups in the PU-SS-COOH-NH_2_-1, PU-SS-COOH-NH_2_-2 and PU-SS-COOH-NH_2_-3 samples results in their charge switchable properties.

### 3.2 Characterization of PU micelles

The synthesized amphiphilic multi-blocked PUs self-assembled in aqueous solution into micelles having a hydrophobic PCL core and a hydrophilic PEG shell, as confirmed by fluorescence measurements using pyrene as a probe (Fig. S2A–D†). The CMCs were also determined by fluorescence spectroscopy with the pyrene probe (Fig. S2E†). The CMCs of PU-SS-COOH, PU-SS-COOH-NH_2_-1, PU-SS-COOH-NH_2_-2 and PU-SS-COOH-NH_2_-3 were determined to be 2.75 × 10^−3^ mg mL^−1^, 2.45 × 10^−3^ mg mL^−1^, 2.4 × 10^−3^ mg mL^−1^ and 2.04 × 10^−3^ mg mL^−1^, respectively.

**Fig. 2:**
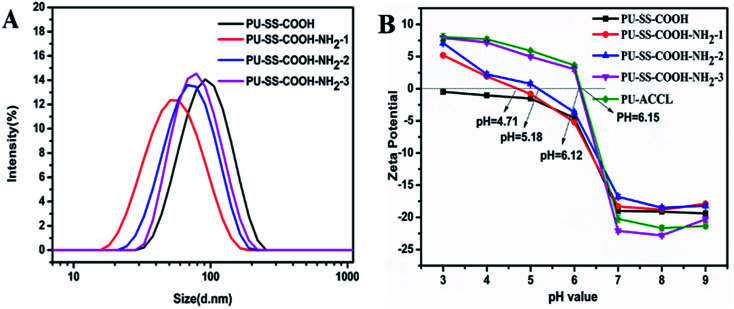
Size distribution of the PU-SS-COOH, PU-SS-COOH-NH_2_-1, PU-SS-COOH-NH_2_-2 and PU-SS-COOH-NH_2_-3 micelles (A). Relationship of pH to zeta potential for PU-SS-COOH, PU-SS-COOH-NH_2_-1, PU-SS-COOH-NH_2_-2, PU-SS-COOH-NH_2_-3 and PU-ACCL micelles (B).

The hydrodynamic diameters of the PU-SS-COOH, PU-SS-COOH-NH_2_-1, PU-SS-COOH-NH_2_-2 and PU-SS-COOH-NH_2_-3 micelles were determined by DLS as 84.5 nm, 48.5 nm, 65.1 nm and 72.7 nm, and the micelles showed unimodal size distributions (Fig. 2A). Notably, the particle sizes of the PU-SS-COOH-NH_2_-1, PU-SS-COOH-NH_2_-2 and PU-SS-COOH-NH_2_-3 micelles were reduced by *ca.* 36, 19.4 and 11.9 nm, respectively, compared with that of the PU-SS-COOH micelles, indicating the successful amidation of the PU-SS-COOH micelles. The size reduction of PU-SS-COOH-NH_2_-1, PU-SS-COOH-NH_2_-2 and PU-SS-COOH-NH_2_-3 micelles compared with the PU-SS-COOH micelles may be ascribed to the enhanced interchain H-bonding among the urethane groups and the amide groups of PU-SS-COOH-NH_2_.

The change of surface charge with solution pH was determined from the relationship of the zeta potential and pH value (Fig. 2B). The presence of both carboxyl and amino groups in PU-SS-COOH-NH_2_-1, PU-SS-COOH-NH_2_-2 and PU-SS-COOH-NH_2_-3 rendered the pH dependant surface charge characteristics of these kinds of PU micelles. All three kinds of PU micelles showed negative zeta potentials at about −20 mV in solutions with a neutral pH or above (pH > 7.0) as a result of the deprotonation of the carboxyl groups in these PU materials; this may facilitate the long blood stream circulation time of these PU micelles.^26^ However, the zeta potential values of all the PU micelles increased with decreasing pH (pH < 6) as a result of the protonation of the primary amine groups and carboxyl groups. Interestingly, as shown in Fig. 2B, the surface charges of PU-SS-COOH-NH_2_-1, PU-SS-COOH-NH_2_-2, PU-SS-COOH-NH_2_-3 and PU-ACCL micelles reversed to become positive at pH values of 4.17, 5.18, 6.12 and 6.15, respectively, due to the protonation of amine groups, which may result in the enhanced cell uptake and the lysosome escape of these two PU micelles (PU-ACCL and PU-SS-COOH-NH_2_-3).^27^

The hydrodynamic diameters of the PU-SS-COOH, PU-SS-COOH-NH_2_-3 and PU-ACCL micelles in Na_2_HPO_4_, as determined by DLS, were 128.8 nm, 103.2 nm and 85.8 nm and they showed unimodal size distributions (Fig. S3A†). Notably, the particle size of the PU-ACCL micelles was reduced by *ca.* 17.4 nm compared with that of the PU-SS-COOH-NH_2_-3 micelles, indicating the successful core-crosslinking of the PU-SS-COOH-NH_2_-3 micelles. As previously reported in the published literature,^28–31^ the particle size of crosslinked polymer micelles is usually found to be smaller than that of their uncrosslinked counterparts due to the more compact chain packing of crosslinked micelles. After they are loaded with drugs, the three kinds of micelles grew larger due to the encapsulation of the hydrophobic drugs into their cores (Fig. S3B†).

**Fig. 3:**
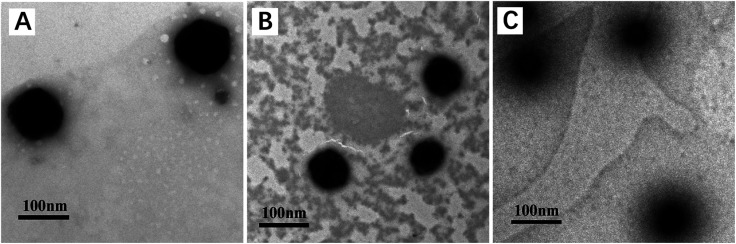
TEM micrographs of PU-SS-COOH (A), PU-SS-COOH-NH_2_-3 (B) and PU-ACCL (C) micelles.

TEM micrographs showed that the assembled PU micelles had spherical morphologies with average diameters of 105.5, 71 and 47 nm for PU-SS-COOH, PU-SS-COOH-NH_2_-3 and PU-ACCL, respectively (Fig. 3A–C). DLS and TEM give different micelle size values due to the dehydrated state of the micelles in TEM.

The pH dependent change in size of the PU micelles is shown in Fig. S4.† The size of PU-SS-COOH-NH_2_ micelles showed an obvious change below pH 5 due to amino protonation in the acidic environment which makes the micelles swell. Compared with the change in size of PU-SS-COOH-NH_2_, a less obvious size change was observed for PU-ACCL due to the crosslinked structures, and the size change of PU-SS-COOH at different pH values was negligible at pH 5–8.

**Fig. 4:**
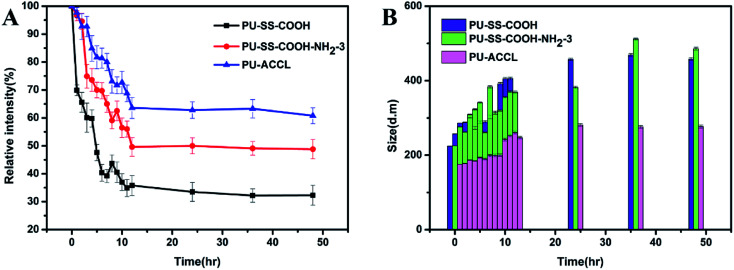
DLS light intensity (A) and size changes (B) of PU micelles in the presence of SDS.

Micelle kinetic stability was studied by using DLS in the presence of SDS, which is a known destabilizing agent. As shown in Fig. 4A, a significant decrease in the scattered light intensity was observed during the first 10 h for SDS-treated PU-SS-COOH and PU-SS-COOH-NH_2_-3 micelles, and the relative intensity was then maintained at 30–45% of the original value. This demonstrated the dissociation of a large percentage of the micelles. In contrast, a drop in scattered light intensity of only 40% was observed for the PU-ACCL micelles in 48 h, indicating the enhanced stability of these PU-ACCL micelles. Fig. 4B shows the size changes of the PU micelles after treatment with SDS, as a function of time. Notably, the size of the PU-ACCL micelles remained stable after treatment with SDS, with an average diameter of less than 300 nm. However, the sizes of the PU-SS-COOH and PU-SS-COOH-NH_2_-3 micelles increased rapidly to about 350 nm or more after treatment with SDS, showing the lower stability of these two kinds of PU micelles.

### 3.3 Drug loading and release

DOX and PTX were used as model hydrophobic drugs to study the loading capacity of the redox and pH active PU micelles using the micelle dialysis method to load the PTX and DOX into the PU micelles. The excess PTX and DOX were removed by filtration through a 0.45 μm filter. The loading levels of PTX in the PTX-loaded micelles were determined with HPLC. The drug-loading content (LC) percentages for PU-SS-COOH, PU-SS-COOH-NH_2_-3 and PU-ACCL PTX-loaded micelles were 10.9%, 12% and 13.65%, respectively, and the encapsulation efficiencies (EE) for PU-SS-COOH, PU-SS-COOH-NH_2_-3 and PU-ACCL PTX-loaded micelles were 24.88%, 27.7% and 32.1%, respectively. The loading levels of DOX in the DOX-loaded micelles were determined using UV spectrophotometry. The LCs for PU-SS-COOH, PU-SS-COOH-NH_2_-3 and PU-ACCL PTX-loaded micelles were 4.98%, 5.11% and 4.44%, respectively, and the EEs for PU-SS-COOH, PU-SS-COOH-NH_2_-3 and PU-ACCL PTX-loaded micelles were 26.3%, 27% and 23.3%, respectively.

**Fig. 5:**
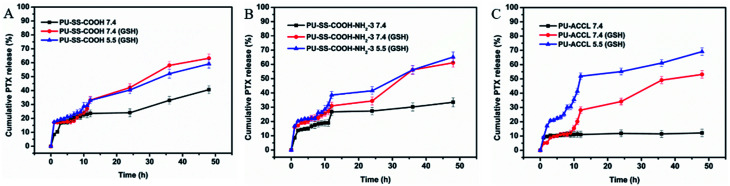
Time dependent cumulative release of PTX from PU-SS-COOH (A), PU-SS-COOH-NH_2_-3 (B) and PU-ACCL (C) micelles in PBS buffer solutions (pH 7.4 or pH 5.5, 10 mM) with or without 10 mM of GSH at 37 °C.

The drug release behaviors of the PTX-loaded PU micelles were investigated in PBS buffer solutions (pH 7.4, 10 mM) with and without 10 mM of GSH at 37 °C. The cumulative drug release profiles as a function of time are plotted in Fig. 5. Without the presence of GSH, an obvious initial burst release was observed for PTX-loaded PU-SS-COOH and PU-SS-COOH-NH_2_-3 micelles, while it was markedly suppressed for the drug-loaded PU-ACCL micelles. For example, only 12% of PTX had been released from the drug-encapsulated PU-ACCL micelles at 48 h in the absence of GSH (Fig. 5C), while 41% and 33% PTX had been released from PTX-encapsulated uncrosslinked PU-SS-COOH and P-SS-COOH-NH_2_-3 at 48 h (Fig. 5A and B). This demonstrated the better stability of PTX-loaded PU-ACCL micelles in PBS than those of the other two drug-loaded uncrosslinked PU micelles. In addition, PTX-loaded P-SS-COOH-NH_2_-3 micelles showed superior stability over PTX-loaded PU-SS-COOH micelles, which is consistent with the CMC results. However, PTX release from all of the three PU micelles was enhanced dramatically when 10 mM GSH was present in the PBS solution, causing 63%, 61% and 53% PTX to be released from PU-SS-COOH, P-SS-COOH-NH_2_-3 and PU-ACCL micelles, respectively, in 48 h. The enhanced drug release in the presence of GSH arose from the disassembly of the PU micelles due to the GSH-induced disulfide cleavage. In an acidic solution (pH 5.5, 10 mM GSH), an obviously enhanced release was observed for PU-ACCL micelles after 7 h when compared with that in neutral solution (pH 7.0, 10 mM GSH), indicating the responsiveness of the Schiff bonds to the acidic environment. In contrast, the release rates were comparable in acidic and neutral environments for PU-SS-COOH and PU-SS-COOH-NH_2_-3.

### 3.4 Internalization and intracellular release of the polyurethane micelle payload

**Fig. 6:**
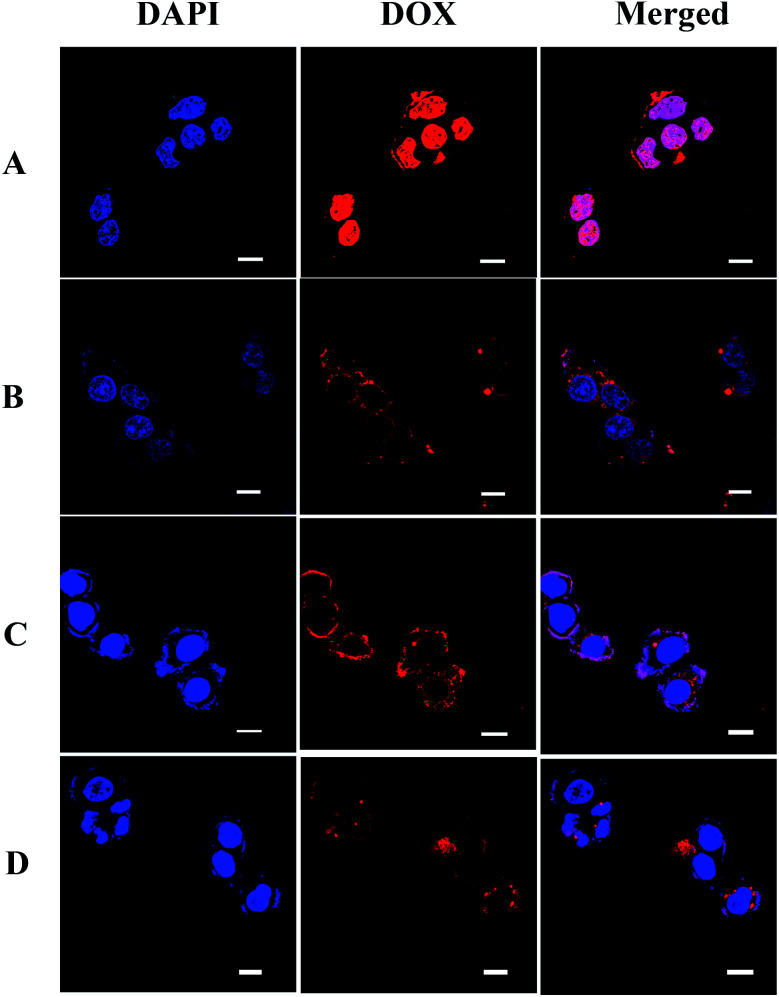
CLSM images of HepG2 cells incubated for 2 h with free DOX (A), PU-SS-COOH (B), PU-SS-COOH-NH_2_-3 (C) and PU-ACCL (D). Scale bar = 10 μm.

CLSM was used to track the cellular uptake and intracellular drug release behavior of DOX-loaded nanoparticles in HepG2 cells. The nuclei of HepG2 cells were stained by DAPI, and presented blue fluorescence which was distinct from the red fluorescence of DOX. Fig. 6 shows CLSM images of HepG2 cells incubated with free DOX and DOX-loaded PU micelles for 2 h. After 2 h of incubation, strong red fluorescence of free DOX was observed in the nuclei, which were even turned pink in the merged fluorescence images as a result of the overlapping fluorescence of DAPI and DOX. Compared with the HepG2 cells incubated with free DOX, a weaker red fluorescence was observed in the cytoplasm and nuclei for DOX-loaded PU micelles, *i.e.* PU-SS-COOH, PU-SS-COOH-NH_2_-3 and PU-ACCL, suggesting the lower internalization efficiency of the carriers. Notably, stronger DOX fluorescence was found for DOX-loaded PU-ACCL micelles than for DOX-loaded PU-SS-COOH micelles, indicating the enhanced internalization of PU-ACCL micelles by HepG2 cells due to their charge switchable properties. The DOX fluorescence images suggest that DOX-loaded PU micelles were internalized and DOX was released, reaching the cell nuclei.

### 3.5 *In vitro* cytotoxicities of polyurethane micelles and DOX-loaded polyurethane micelles

The *in vitro* cytotoxicities of drug-free and drug-loaded PU micelles were evaluated using the CCK8 assay. The results showed that empty PU-SS-COOH, PU-SS-COOH-NH_2_-3 and PU-ACCL micelles had very low cytotoxicity (greater than 80% cell viability) in two different cell lines, even at micelle concentrations of up to 1 mg mL^−1^, suggesting the nontoxic nature of the PU micelles to HUVEC and HepG2 cells (Fig. S5†).

**Fig. 7:**
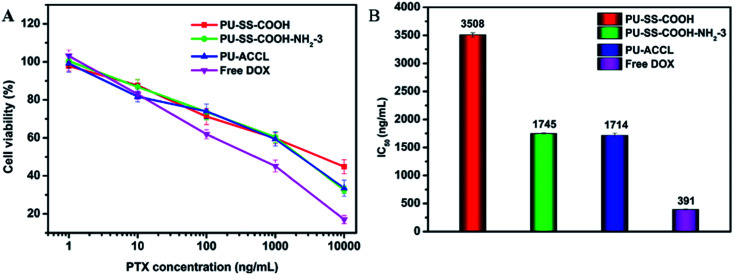
Cytotoxicity (A) and IC_50_ values (B) of DOX-loaded PU micelles against HepG2 cells after incubation for 24 h.

Further, the *in vitro* cytotoxicities of DOX-loaded PU-SS-COOH, PU-SS-COOH-NH_2_-3 and PU-ACCL micelles and free DOX is shown in Fig. 7. Fig. 7A shows the cytotoxicity results given as a function of DOX concentration from 1 to 10 000 ng mL^−1^. The IC_50_ (*i.e.*, inhibitory concentration to produce 50% cell death) values of DOX-loaded PU-SS-COOH, PU-SS-COOH-NH_2_-3 and PU-ACCL micelles, and free DOX were determined to be ∼3508, ∼1745, ∼1714 and ∼391 ng mL^−1^, respectively, for HepG2 cells. The results reveal that the PU-SS-COOH-NH_2_-3 and PU-ACCL micelles provided higher efficiency intracellular delivery of DOX as compared to PU-SS-COOH micelles, due to the protonation of amine groups and the charge switchable properties in the acidic environment.

## 4. Conclusion

In summary, redox sensitive PU micelles with tunable pH induced surface charge switchabilities were core crosslinked with acid cleavable Schiff bonds, and were investigated as anticancer drug carriers. The crosslinked reduction responsive biodegradable micelles show superior stability with negative surface charges in normal physiological conditions (pH = 7.5, without GSH). This is favorable for long circulation times and low levels of premature release of drugs. When located at the sites of acid-associated tumor tissues, the primary amine groups are protonated, yielding a net positive zeta potential on the surface of these PU-ACCL micelles, which facilitates the penetration of the PU micelles into deep cancer tissues and tumor cells. After entry into tumor cells, the intracellular GSH and acidity causes the rapid disassembly of PU-ACCL as a result of reduction/pH induced cleavage of disulfides/Schiff base bonds, which is favorable for rapid drug release and endosome escape. Therefore, the core crosslinked surface charge switchable PU micelles with reduction and pH responsive properties may hold great potential for a bio-triggered drug delivery system for cancer therapy.

## Conflicts of interest

There are no conflicts to declare.

## Supplementary Material

RA-008-C8RA01581C-s001
