# Supplementary material for: Reduction responsive and surface charge switchable polyurethane micelles with acid cleavable crosslinks for intracellular drug delivery
Source: RSC Adv. 2018 May 16;8(32):17888–97. doi: 10.1039/c8ra01581c (PMC9080465; doi:10.1039/c8ra01581c)
Supplement: RA-008-C8RA01581C-s001 [file RA-008-C8RA01581C-s001.pdf]

Electronic Supplementary Material (ESI) for RSC Advances.
This journal is © The Royal Society of Chemistry 2018

Supporting Information

Reduction responsive and surface charge switchable polyurethane micelles with acidity cleavable crosslinks for intracellular drug delivery

Lili Zhao, Chang Liu, Zhuangzhuang Qiao, Yan Yao and Jianbin Luo*

College of Chemistry and Environmental Protection Engineering, Southwest Minzu University,

610041 Chengdu, China

Tel: +86 28 6902063. Fax: +86 28 85522322

E-mail addresses: luojb1971@163.com

Supplemental Figures

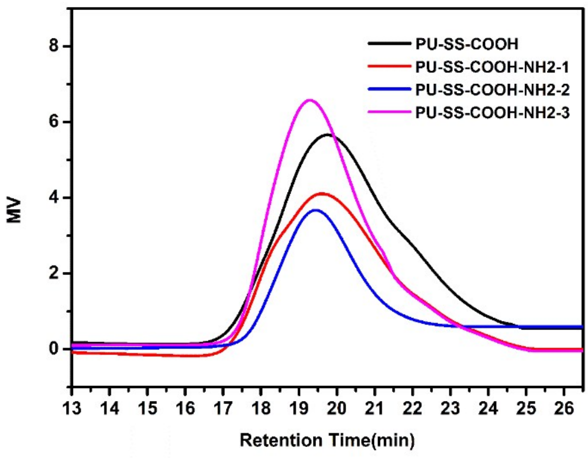

Figure. S1. The GPC curves of PU-SS-COOH, PU-SS-COOH-NH$_2$-1, PU-SS-COOH-NH$_2$-2 and PU-SS-COOH-NH$_2$-3.

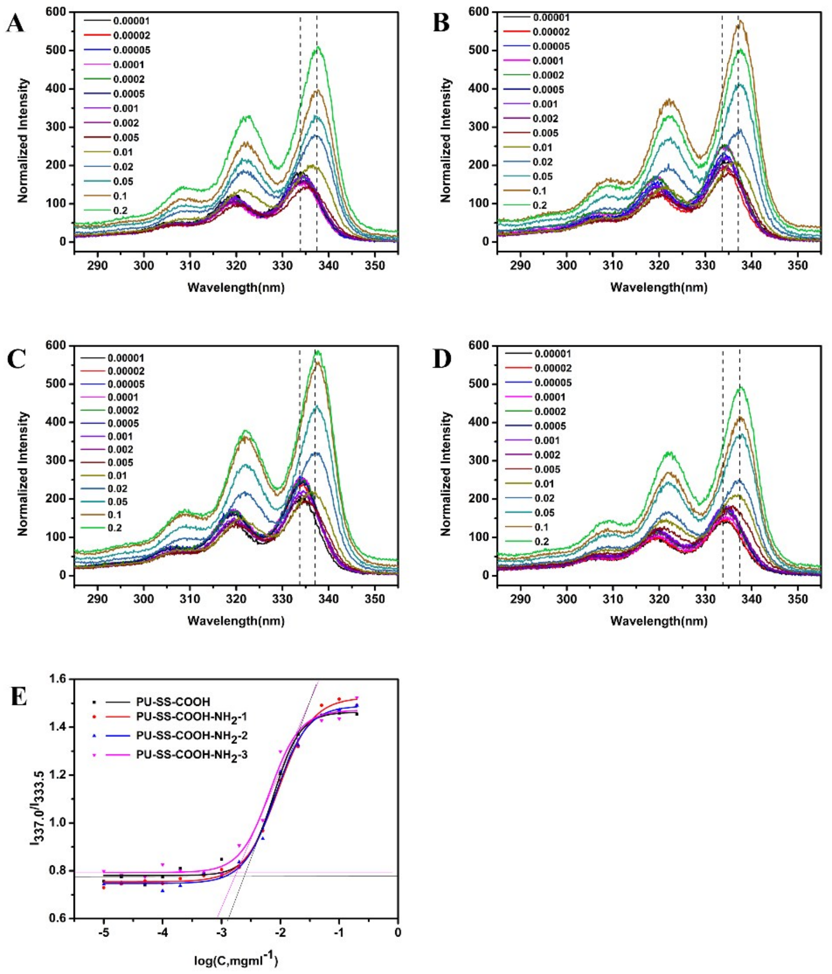

Figure. S2. Typical fluorescence excitation spectra (372 nm) of PU-SS-COOH (A), PU-SS-COOH-NH_2 -1 (B), PU-SS-COOH-NH_2-2 (C) and PU-SS-COOH-NH_2 -3 (D). $I_{337.0}/I_{333.5}$ ratios in the excitation spectra as a function of micellar concentrations (log C.mg.ml^{-1}). The CMC was obtained from the intersection of the two tangent lines shown by the arrows. The CMC of PU-SS-COOH, PU-SS-COOH-NH_2-1, PU-SS-COOH-NH_2-2 and PU-SS-COOH-NH_2-3 was determined to be 2.75×10^{-3} mg.ml^{-1} , 2.45×10^{-3} mg.ml^{-1}, 2.4×10^{-3} mg.ml^{-1} and 2.04×10^{-3} mg.ml^{-1}. (E)

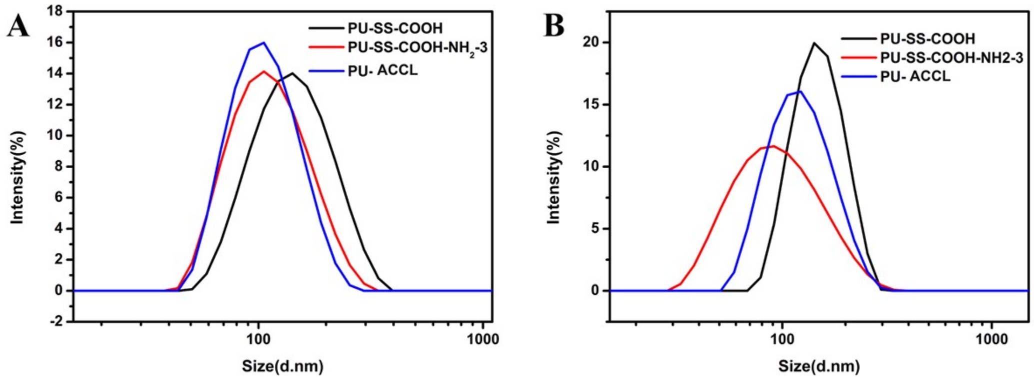

Figure. S3. Size distribution of the PU-SS-COOH, PU-SS-COOH-NH_2-3and PU-ACCL micelles exist in Na_2HPO_4(A) and their PTX loaded micelles (B).

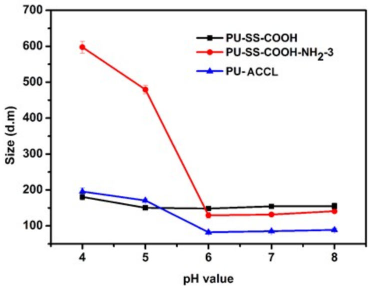

Figure. S4. pH dependent size changing of of PU micelles.

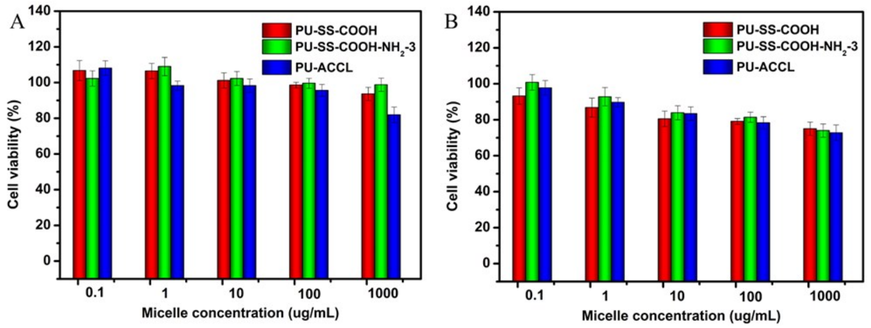

Figure. S5. Viability of HUVECs cells (A) and HepG2 (B) after 48 h of incubation with various concentrations of empty reduction-sensitive polyurethane micelles determined by the CCK8 assay.
